# Identifying autism spectrum disorder symptoms using response and gaze behavior during the Go/NoGo game CatChicken

**DOI:** 10.1038/s41598-021-01050-7

**Published:** 2021-11-10

**Authors:** Prasetia Utama Putra, Keisuke Shima, Sergio A. Alvarez, Koji Shimatani

**Affiliations:** 1 Graduate School of Engineering, Yokohama National University, Yokohama, Japan; 2 Faculty of Engineering, Yokohama National University, Yokohama, Japan; 3 Department of Computer Science, Boston College, Chestnut Hill, USA; 4 Faculty of Health and Welfare, Prefectural University of Hiroshima, Hiroshima, Japan

**Keywords:** Signs and symptoms, Information technology

## Abstract

Previous studies have found that Autism Spectrum Disorder (ASD) children scored lower during a Go/No-Go task and faced difficulty focusing their gaze on the speaker’s face during a conversation. To date, however, there has not been an adequate study examining children’s response and gaze during the Go/No-Go task to distinguish ASD from typical children. We investigated typical and ASD children’s gaze modulation when they played a version of the Go/No-Go game. The proposed system represents the Go and the No-Go stimuli as chicken and cat characters, respectively. It tracks children’s gaze using an eye tracker mounted on the monitor. Statistically significant between-group differences in spatial and auto-regressive temporal gaze-related features for 21 ASD and 31 typical children suggest that ASD children had more unstable gaze modulation during the test. Using the features that differ significantly as inputs, the AdaBoost meta-learning algorithm attained an accuracy rate of 88.6% in differentiating the ASD subjects from the typical ones.

## Introduction

People often misinterpret invisible disorder symptoms in their children, such as inattentiveness and difficulty communicating with other people, as willful misconduct or misbehavior. The prevalence of clinical disorders, however, is high. In Japan, the prevalence of Autism Spectrum Disorder (ASD) symptoms among children has been estimated to be between 1.9 and $9.3\%$ based on parent and teacher reports ^1^; in the USA, about 1 of 54 children was diagnosed with ASD in 2020 ^2^, while in 2016, a study found that 9.41% of children had Attention Deficit Hyperactivity Disorder (ADHD) symptoms^3^.

Since the conventional diagnosis method requires comprehensive tests that are time-consuming, many studies have proposed to automatically distinguish disordered children from typical ones by utilizing machine learning with biosignals such as EEG^4^ or fMRI^5^. Those methods extracted features from children’s brain activity to identify disorder symptoms.

In contrast, psychiatry studies recognize disorder symptoms by employing behavioral tests, e.g., Go/NoGo^6–8^ and visual attention tests. Previous studies have discovered a significant difference between ASD and typical children during a Go/NoGo task. The task requires a subject to react to the Go stimulus and inhibit their reaction to the NoGo stimulus^8^. The stimuli can be represented by visual objects with different colors and shapes^6,8^ or by sounds with different frequencies^7^. The task evaluates the subjects by measuring their percentages of correct responses and omission errors and their average response time and its variability. Children with ASD tend to perform worse^9^ and have high response time variability^10^ than typical children during the task, which may be caused by variability in neural activations^11^.

Moreover, studies on ASD children’s gaze behavior have observed that the ASD group was slower to adjust their gaze to the stimulus position during eye-tracking measurement of joint attention^12^, and faced difficulty in modulating their gaze during face-to-face conversation^13^. Previous works have found that temporal features of gaze are more informative than global measurements in differentiating ASD from typically developing children. Swanson and Siller^12^ have found that ASD and typical children allocated the same amount of time to key areas but their duration of the first fixation to the target differed. Likewise, studies of gaze-shift^14^ and gaze-to-stimulus-distance^15^ have signified that gaze behavior of ASD and typical children differed significantly in the spatio-temporal aspect.

Other researchers extend those works by employing machine learning, and children’s behavior features to identify ASD symptoms^16,17^. They asked participants to participate in face-to-face conversation^18^ or to complete visual tasks such as viewing a sequence of face images^16^ or identifying directional cues^17^. Then they utilized spatial features extracted from children’s eye movement distribution to recognize ASD symptoms in children.

This study aims to investigate the response and gaze behavior of children during the Go/NoGo task and to utilize features extracted from those measurements to identify ASD symptoms that suggest difficulty in inhibiting action and point of view^19^. Contrary to previous works on ASD subjects’ gaze behavior, which have focused on global summary measures related to the gaze and stimulus positions, this study examined the intrinsic spatio-temporal structure of the gaze trajectories in greater detail by employing entropy-based and autoregressive features.

Using the CatChicken game^20^, we measured 21 ASD (10 with and 11 without ADHD) and 31 typical children’s response and gaze modulation; the use of a standardized task minimizes the bias that often occurs in face-to-face conversation. Spatial and gaze-adjustment features were extracted to represent each child’s response, performance during the game, and gaze behavior. Statistical comparisons between typical and ASD disorder children were performed using Student *t* and Mann-Whitney *U* tests^21^. Additional details of the statistical methodology appear in the Methods section. The AdaBoost algorithm was employed^22^ to distinguish the features of ASD disorder children from those of typical children. Experiments employing spatial features, gaze-adjustment features, and a combination of them were conducted to identify differentiating features. Classification performance of the model was evaluated with accuracy, Matthews Correlation Coefficient (MCC)^23^, and Area Under the Curve (AUC)^24^ metrics and validated using three-fold cross-validation.

## Results

### Spatial features

Statistical analysis demonstrated a significant difference (by both Student *t* and Mann-Whitney *U* tests) between typical and ASD groups for eight spatial features ($n = 52$, $p < 0.01$ corrected by Benjamini-Hochberg at the level 0.05; see Supplementary Materials for further detail): variance of fixation time, average and entropy of gaze acceleration, spectral entropy of gaze-to-object-distance, sample entropy of gaze distance, gaze angle, gaze-to-obj-distance, and velocity. For all such variables, the mean of the second group (ASD) was larger than that of the first group (typical); the corresponding effect sizes were large^25^ ($|d| > 0.8$), except for average acceleration, for which a moderately large effect size ($d = -0.763$) was observed.

In contrast, although a medium effect size ($|d| > 0.5$) was observed for Go positive and negative percentages, response-time variance (RT-var), and gaze-acceleration standard deviation, the differences between typical and ASD groups for those variables were insignificant. Nevertheless, within-group mean values indicated that ASD children more often responded incorrectly with higher response-time variance than the typical subjects.

A comparison between typical and ASD children without ADHD yielded similar results. The results, however, also suggested a significant difference in Go positive and negative percentage between those groups ($n = 42$, $p < 0.008$). Typical children responded correctly towards the Go stimulus more often than ASD participants without ADHD ($d > 0.99$). A significant difference in RT-var (Mann-Whitney $p = 0.012$) was observed between those groups.

Different results were observed in the statistical comparison between typical and ASD subjects with ADHD. An insignificant difference ($n = 41$, $p > 0.02$) was observed for spectral entropy of gaze-to-object-distance. The Student *t*-test suggested a significant difference ($p < 0.012$) of gaze acceleration variance between those groups. The statistical tests also showed that ASD children without ADHD did not differ from the children with ADHD ($n = 21$, $p > 0.09$). However, the latter had a lower gaze-fixation time than the former (moderate effect size, $d = 0.726$).

Similarly to the results of comparison between typical and ASD groups described above, statistical analysis by ANOVA revealed that typical and ASD participants with and without ADHD differed significantly in the same eight features($n = 52$, $p < 0.02$). The results also showed a significant difference in gaze acceleration variance ($p = 0.003$) and in the average and variance of gaze distance and velocity ($p <= 0.025$).

### Gaze-adjustment features

A significant difference ($n = 208$, $p < 0.023$) between the groups was observed in the mean values of $\alpha$, $\theta _1$ and $\theta _2$ by the Mann-Whitney *U* test. In contrast, both the Student *t*-test and effect size ($|d| < 0.2$) suggested that ASD children’s gaze-adjustment features did not differ from the typical ones.

Separating gaze-adjustment features according to response types (Go-positive, Go-negative, NoGo-positive, and NoGo-negative) yielded statistically significant differences ($n = 52$, $p < 0.023$) between typical and ASD children in all auto-regressive coefficients by the Mann-Whitney *U* test, as well as greater effect size (mean |*d*| > 0.4). The *t*-test results also signified that ASD gaze modulation differed when they responded incorrectly to the Go stimulus and correctly to the NoGo stimulus ($p < 0.007$).

**Figure 1:**
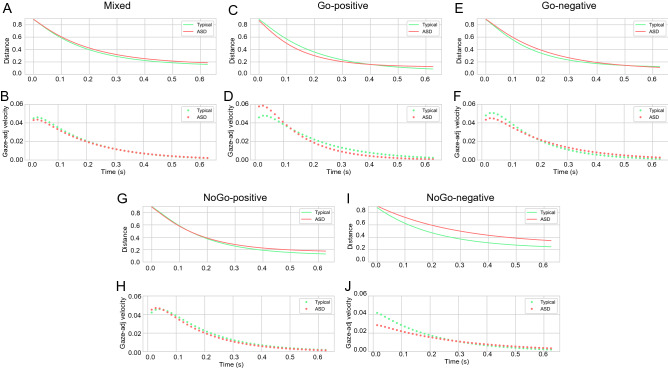
Extrapolating results of Auto-regressive model using the average of parameters. Gaze extrapolation results using mixed (**A**), Go positive (**C**) and negative (**E**), and NoGo positive (**G**) and negative (**I**) coefficients. (**B,D,F,H,J**) show respectively the extrapolated gaze-to-obj distance and velocity results for mixed (typical-avg: 0.0161, ASD-avg: 0.0156), Go positive (typical-avg: 0.0178, ASD-avg: 0.0165) and negative (typical-avg: 0.0169, ASD-avg: 0.0173), and NoGo positive (typical-avg: 0.0170, ASD-avg: 0.0158) and negative (typical-avg: 0.0141, ASD-avg: 0.0124) coefficients. Solid and dotted green lines represent, respectively, typical children’s extrapolated gaze-to-obj distance and the negative of its first derivative (gaze-adjustment velocity) over time. ASD children’s extrapolated gaze-to-obj distance and gaze-adjustment velocity are represented by sold and dotted orange lines, respectively.

Furthermore, extrapolation of the gaze-to-obj distance in time using the average values of the autoregressive coefficients suggested that separating the features (Fig. 1C–J) produced a more obvious difference between the groups than mixing them (Fig. 1A,B). Typical children adjusted their gaze to the stimulus position faster when they responded correctly to the Go and NoGo characters and when they reacted incorrectly to the latter stimulus (Fig. 1C,G,I); the velocity of their extrapolated gaze-adjustment (Fig. 1D,H,J) was ±0.0014 faster compared to the ASD children (the velocity of extrapolated gaze-adjustment was computed by averaging the negative of the first derivative of the extrapolated gaze-to-obj distance over time). Nevertheless, typical children modulated their gaze in a similar way to the ASD subjects when they missed the Go stimulus (Fig. 1E,F).

The Student *t*-test between the typical and ASD children without ADHD suggested those groups differed when they responded correctly towards the Go and NoGo stimuli ($n = 52$, $p <= 0.004$). Comparison results of typical children to ASD children with ADHD indicated that the former responded differently from the latter during Go-negative and NoGo-positive ($n = 52$, $p <= 0.017$). The results also demonstrated that ASD children with and without ADHD did not differ significantly. Moreover, the ANOVA test showed a significant difference ($n = 52$, $p < 0.04$) among those three groups for gaze-adjustment features when the subjects responded correctly to the NoGo stimulus; the difference was insignificant in the other conditions.

**Figure 2:**
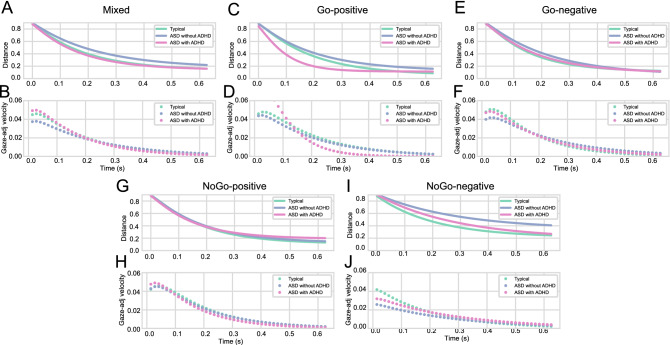
Extrapolating results of Auto-regressive model using the average of parameters. Gaze extrapolation results using mixed (**A**), Go positive (**C**) and negative (**E**), and NoGo positive (**G**) and negative (**I**) coefficients. (**B,D,F,H,J**) show respectively the extrapolated gaze-to-obj distance and velocity results for mixed (typical-avg: 0.0161, ASD without ADHD-avg: 0.0150, ASD with ADHD-avg: 0.0160), Go positive (typical-avg: 0.0178, ASD without ADHD-avg: 0.0162, ASD with ADHD-avg: 0.0162) and negative (typical-avg: 0.0169, ASD without ADHD-avg: 0.0175, ASD with ADHD-avg: 0.0170), and NoGo positive (typical-avg: 0.0170, ASD without ADHD-avg: 0.0165, ASD with AD-avg: 0.0152) and negative (typical-avg: 0.0141, ASD without ADHD-avg: 0.0111, ASD with ADHD-avg: 0.0140) coefficients. Solid and dotted green lines represent, respectively, typical children’s extrapolated gaze-to-obj distance and the negative of its first derivative (gaze-adjustment velocity) over time. Extrapolated gaze-to-obj distance and gaze-adjustment velocity of ASD children with and without ADHD symptoms are represented by purple and pink colors, respectively.

The extrapolation results of ASD children with and without ADHD symptoms (Fig. 2A–J) suggested that the former tended to adjust their gaze to the stimulus position slightly faster than the latter, with respective extrapolation gaze-adjustment velocities of 0.0153 and 0.0156, respectively. Both groups had lower gaze-modulation speed compared to typical participants, whose average velocity was 0.0164.

### Classification

Classification results (Table 1) showed that when using only spatial features, the accuracy of the AdaBoost model was 6.1% lower than when utilizing gaze-adjustment features. A significant increase in the model’s recognition rate occurred when separating the gaze-adjustment features based on response types (response-type-gaze features). The classification rate was 17.1% higher than when employing gaze-adjustment features.

**Table 1:** The AdaBoost algorithm obtained high performance when employing gaze-related features.

Modality	Accuracy (mean ± S.D.)	MCC (mean ± S.D.)	AUC (mean ± S.D.)
Spatial	65.1 ± 8.9	0.29 ± 0.17	0.67 ± 0.09
Gaze-adjustment	71.2 ± 7.9	0.40 ± 0.16	0.79 ± 0.11
Response-type-gaze	88.3 ± 4.9	0.79 ± 0.08	0.95 ± 0.02
Response-type-gaze+significant	88.6 ± 4.4	0.80 ± 0.07	0.90 ± 0.03
Response-type-gaze+significant+performance	84.4 ± 10.1	0.71 ± 0.19	0.90 ± 0.01

Accuracy rate, MCC, and AUC scores of the AdaBoost algorithm with different modality as inputs (mean ± S.D.). “Significant” refers to features that differ significantly between the groups. “Performance” denotes game performance features that includes positive and negative Go and NoGo responses, response time, and response time variability.

Using both response-type-gaze and significant spatial features, the model obtained an insignificant increase in its accuracy rate, which was 0.3% higher than when using the former features alone. Combining the gaze features with significant spatial and game performance features, however, decreased the accuracy rate by 4.1%.

The MCC score agreed with the accuracy results: combining response-type-gaze features and significant spatial features yielded a 0.01 higher MCC score than using only response-type-gaze features. Even though the increase was insignificant, the high MCC score indicated that the model’s prediction results strongly correlated with the ground-truth labels, and the model could reliably recognize both the typical and ASD children. Besides, the AdaBoost obtained AUC scores higher than 0.85 when utilizing response-type-gaze features and when combining them with significant spatial and performance features. This suggested that high performance could be expected from the algorithm when employing those features^26^.

**Figure 3:**
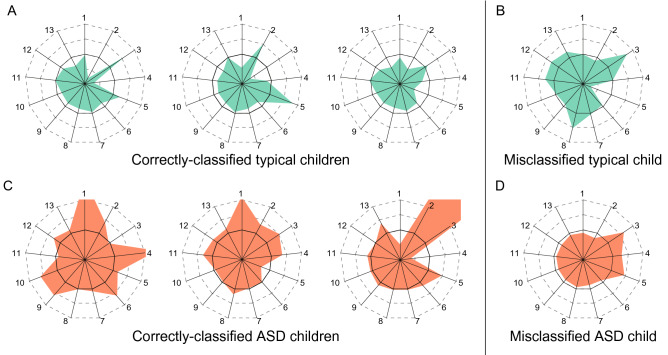
Star plots depicting four subjects’ response-type-gaze and significant spatial features.(**A**,**B**) Features representing correctly-classified and misclassified typical children. (**C**,**D**) Features representing correctly-classified and misclassified ASD children. Black-line indicates zero values. 13 indexes represent reduced gaze-adjustment features (1–5), velocity-sen, acceleration-avg, fixation-var, distance-sen, angle-sen, gaze-obj-en, gaze-obj-sen, and gaze-obj-spe.

The confusion matrix (Table 2), however, shows that the model more frequently misclassified typical subjects as ASD (false-positive) than it misclassified ASD subjects as typical (false-negative). Visualization of the features through star plots (Fig. 3) reveals higher mean values and variability in the misclassified typical subjects’ features than the correctly-classified subjects’. On the other hand, the features of misclassified ASD subjects show lower mean values and variability.

**Table 2:** False-positive rate of the AdaBoost algorithm was higher than false-negative rate.

	Predicted: typical (%)	Predicted: ASD (%)
Actual: typical	83.9	16.1
Actual: ASD	4.8	95.2

Confusion matrix of classification results using response-type-gaze and significant spatial features as input of the AdaBoost algorithm.

In differentiating the typical group from ASD children with and without ADHD using gaze-adjustment and significant spatial features (Table 3), the AdaBoost algorithm achieved a 17.5% lower accuracy rate than when classifying typical and ASD populations. Also, combining the gaze-adjustment features with significant features selected by ANOVA resulted in a 30.9% lower recognition rate. The MCC scores and the confusion matrix results (Table 4) suggested that the model had poor performance in recognizing ASD children with and without ADHD symptoms.

**Table 3:** Although the AdaBoost algorithm achieved a competitive accuracy rate, its low MCC score indicated high false positive and negative.

Modality	Accuracy (mean ± STD)	MCC (mean ± STD)	AUC (mean ± S.D.)
Response-type-gaze+significant	71.0 ± 5.3	0.449 ± 0.118	0.758 ± 0.098
Response-type-gaze+significant (ANOVA)	57.6 ± 7.6	0.180 ± 0.102	0.659 ± 0.113

Accuracy rate, MCC, and AUC scores of the AdaBoost algorithm in recognizing ASD and ASD with ADHD symptoms in children. “ANOVA” indicates that the significant spatial features are determined by referring to *p*-value of ANOVA.

**Table 4:** Misclassification of ASD populations were higher than that of typical subjects.

	Predicted: typical (%)	Predicted: ASD without ADHD (%)	Predicted: ASD with ADHD (%)
Actual: typical	96.8	0.0	3.2
Actual: ASD without ADHD	54.5	18.2	27.3
Actual: ASD with ADHD	30.0	20.0	50.0

Confusion matrix of classification results using response-type-gaze and significant spatial features as input of the AdaBoost algorithm in differentiating typical from ASD subjects with and without ADHD.

## Discussion

This study evaluated whether features extracted from response and gaze behavior during Go/NoGo task can be used to identify ASD symptoms in children. We utilized the CatChicken game^20^ to measure the response and gaze modulation of 21 ASD and 31 typical children. During the game, the children should respond to the chicken character (Go stimulus) by pressing a space bar but should inhibit their action towards the cat character (NoGo stimulus).

The game outputs four variables: response types and times, and the stimulus and gaze locations over time. Statistical analyses using Student *t* and Mann-Whitney *U* tests were performed on spatial and gaze-adjustment features extracted from those variables.

As we expected, we found a significant difference in gaze modulation between ASD and typical children. Previous studies found that ASD children’s gaze movement differed significantly from typical children’s in terms of variability of the gaze pattern^27^, the fixation time spent on the stimulus^13^, and duration of the first fixation to the target^12^. Our results suggest lower accuracy and greater randomness of the ASD subjects’ visual tracking of the target: the relative gaze-to-object difference was less steady over time than for typical subjects, and predictability of ASD subjects’ gaze was lower as measured by sample entropy of both distance and angle. A greater irregularity of gaze distance and angle may indicate that ASD children over-interpreted the information of a given stimulus, thereby causing more unintentional viewing behavior^28^. The higher value of ASD children’s gaze-to-object entropy suggested less structured tracking in a spatial sense, while a greater value of sample entropy value demonstrated lowered predictability of the gaze-to-object difference as a function of time. Likewise, greater spectral entropy indicated less structure of the frequency content of ASD subjects’ gaze signals.

Compared to typical subjects, we observed that ASD subjects without ADHD symptoms tended to perform worse while the children with ADHD had less structured gaze modulation. Nevertheless, the results demonstrated that the game performance of ASD subjects without ADHD did not differ significantly from that of the subjects with ADHD symptoms. We found that the ASD children without ADHD symptoms tended to fixate their gaze on the stimulus position longer than the children with ADHD symptoms.

Second, statistical analysis using the Mann-Whitney *U* test demonstrated significant differences in the gaze-adjustment features among the groups. The extrapolation results show that children with ASD symptoms, on average, adjusted their gaze more slowly to the stimulus location than their typical peers. When ASD children reacted incorrectly towards the stimulus, their extrapolation results tended to be slower at the beginning; these results were also observed when comparing the ASD group with ADHD to the group without it. The results, however, did not signify that typical subjects’ gaze movement was faster, as an insignificant difference in gaze velocity was observed between the groups.

In contrast, gaze trajectory area and the entropy of gaze distribution showed no significant differences between typical and ASD populations. While our previous work^20^ found greater dispersion of gaze movement in ASD children, the results of the present paper suggest that global measures of gaze behavior are similar in the two groups. The discrepancy may be due to the greater size of the sample available for the present paper. Swanson and Siller^12^ also observed that total gaze allocation of ASD and typical children did not differ but their temporal gaze movement (duration of the first fixation to the target) did. Our present findings and theirs provide compelling evidence that the gaze behavior of ASD children may differ from the typically developing children in the temporal aspect; the difference was more pronounced between typical and ASD children with ADHD symptoms.

Another major finding of this study was that the performance of the game and response time of typical and ASD children did not differ significantly. Even though we observed greater Go and NoGo negative percentages and higher response time variance in ASD children, statistical analyses demonstrated an insignificant difference between the groups. The results contradict previous works that observed greater RT variability of go-response^9^ and higher omission error^10^ in ASD population than their typical peers. One interpretation of these findings is that the insignificant difference of RT occurred because this work computed RT of both Go and NoGo trials; Lee *et.al*^29^ found similar RT for ASD and typical subjects. Outlier removal in the pre-processing step of the present work might affect the statistical results of game performance and RT variability, as well. Nevertheless, we observed that the ASD group without ADHD had a lower Go-positive score and higher response time variance than typical subjects.

Lastly, our classification results suggest the promising performance of more detailed spatio-temporal features extracted from children’s gaze during the Go/NoGo task. Compared to previous works utilizing global features^16,17^, our model yielded competitive results. Nevertheless, the accuracy rate of our model was lower than that of the previous work utilizing features from visual fixation and session length^18^. The discrepancy might be affected by different features, experiment protocol, and subjects used in the previous study. Furthermore, our classification results show that using responses and gaze features together produced a higher recognition rate in differentiating ASD from typical children than using either type of information alone, as indicated by high accuracy rate, MCC, and AUC scores. The results, however, revealed that a promising performance could not be achieved to identify ADHD symptoms in ASD children. The results might be affected by unbalanced labels: the training data of each fold comprised 58.8% typical, 20.6% ASD with ADHD, and 20.6% ASD without ADHD.

Two limitations of this work are the relatively small sample size and the limited number of features. Also, since this work only measured response behavior by calculating response time and game performance, which represented the execution stage of response, the difference between groups in the preparation stage of response is unclear. Future studies should measure both the preparation and execution stages of participants’ responses. It would be of interest to consider subjects across a broader age range to enable capturing a greater variety of behaviors. This study involved 22 ASD and 35 typical children with a narrow age range and found that among these subjects only one gaze modulation existed: all subjects adjusted their gaze to the stimulus position. In contrast, the results of our previous work^20^ suggested that older subjects were of two types by viewing behavior: ones who adjusted their gaze (55.9% of total subjects) and ones who concentrated on the middle of the screen (44.1% of total subjects).

## Conclusion

This study examined the difference in gaze behavior and response features of ASD and typical children during the Go/NoGo task. Contrary to our hypothesis, the experimental results of this paper showed higher performance in differentiating ASD from typical children using gaze behavior alone, as compared with a combination of gaze behavior features with features extracted from participants’ responses. Even though the use of the features showed promising performance in identifying ASD symptoms, it yielded poor performance in identifying ADHD symptoms in ASD children.

## Methods

### CatChicken game

**Figure 4:**
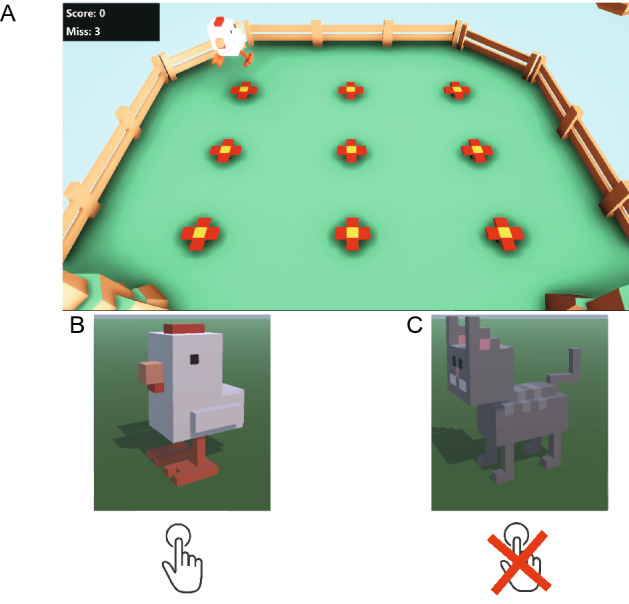
Game interface of the CatChicken system. (**A**) Nine red flowers representing the locations in which a stimulus can appear; (**B**) Go and (**C**) NoGo characters.

The CatChicken^20^ game was utilized to measure children’s response and gaze movement during a Go/NoGo task. The Go/NoGo task was used to measure a person’s inhibitory control; a subject should respond to the Go stimulus but inhibit their action towards the NoGo stimulus^8^. The game represented the Go and NoGo stimuli as “Chicken” and “Cat” characters, respectively. A stimulus appeared randomly in one of nine locations for a fixed duration of time (Fig. 4). The interval between two consecutive stimuli was set by configuring the minimum and maximum waiting-time values.

**Figure 5:**
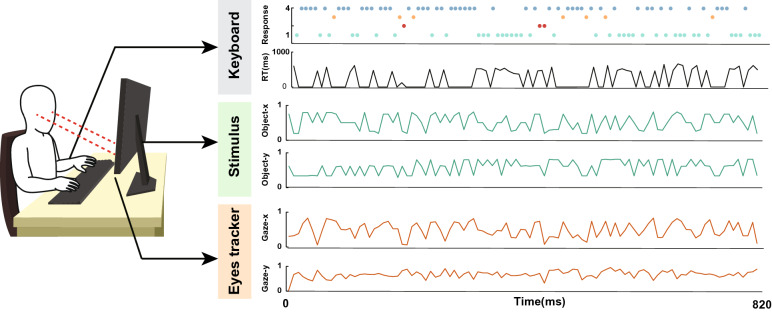
Information measured by the CatChicken system. While playing the Go/NoGo game, CatChicken records children’s response types and times, and locations of stimulus and gaze over time. The response types are Go-positive (green), NoGo-positive (blue), Go-negative (orange), and NoGo-negative (red). The values of object and gaze locations are normalized to range from 0 to 1.

The system outputted the user’s response types and time, and stimulus and eye locations on the monitor (Fig. 5). A user responded to the stimulus by pressing the spacebar. The system categorized a subject’s response as one of four types: Go-positive if the subject responded to the Go character; Go-negative if they missed it; NoGo-positive if they inhibited their action in response to the NoGo character; NoGo-negative if they reacted to it. Different audio feedback was given when the subject responded correctly and incorrectly towards the stimulus. The system was equipped with a Tobii 4C eye tracker that recorded the user’s eye position on the monitor continuously. The eye tracker sampling rate was 90 Hz (interlaced), and its operating distance was 50 cm to 95 cm. The stimulus and eye locations on the monitor were normalized to the unit interval [0, 1] by dividing the pixel coordinates by the window’s coordinate length.

### Participants

Participants involved 22 autism spectrum disorder children (16 male and 6 female) and 35 typical children (24 male and 11 female) with an average age of five years from two local schools in Japan (Table 5). All ASD subjects attended special education school and had been diagnosed by clinicians; 10 (+ one suspected) ASD children also had attention deficit symptoms, and seven of them were identified as having hyperactivity as well. Both ASD and typical children did not have any physical disorder. One ASD child (male) and four typical children (1 male and 3 female) were excluded because their data were corrupted. Therefore, this study only processed 21 ASD and 31 typical children’s data.

**Table 5:** Differences for age and Development Quotient (DQ) were insignificant ($p > 0.05$).

	Male/Female	Age in years (Mean ± STD)	Mean of DQ scores (Mean ± STD)
Typical	24/11	5.0 ± 0.6	96.1 ± 3.0
ASD	16/6	4.6 ± 0.4	95.7 ± 10.4
Student *t*-test	–	0.234	0.913

The average and standard deviation (STD) of age and DQ score of typical and ASD groups. All children participated in this study were Japanese.

**Figure 6:**
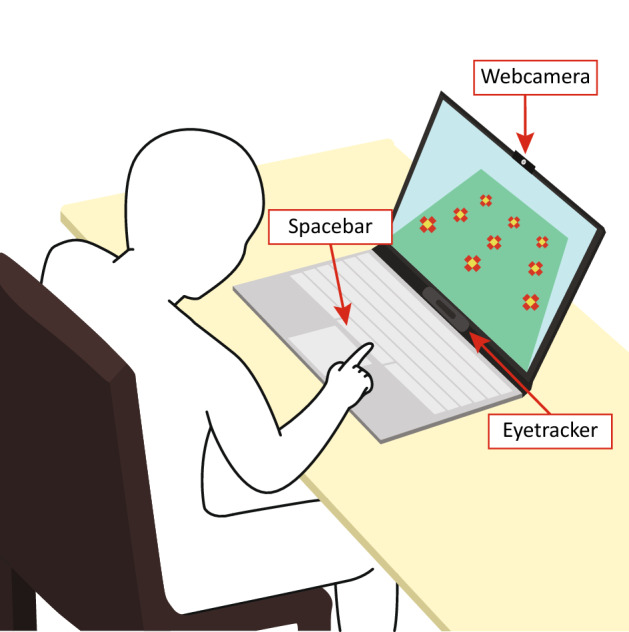
Experimental protocol of this study. The distance between the child and the monitor was about 60 cm. The notebook was equipped with a web camera and an eye tracker.

During the experiment, the subjects were seated in front of a notebook equipped with an eye tracker and web camera (Fig. 6). They responded to the stimulus by pressing the spacebar on the keyboard. The proportion of the Go and NoGo stimuli was uniform and the order of appearance was set in advance; their appearance time was 700 ms; the minimum and maximum of the waiting period were 700 and 1000 ms.

Before starting the experiment, the eye tracker was calibrated and an instructor explained the game and its rules to the subject; the instructor asked the subjects to respond immediately when the stimulus appeared. All subjects participated in a one-minute training session before taking a four-minute evaluation.

#### Ethics statement and consent

Before participating in the experiment, informed consent was obtained from teachers and parents on behalf of the children. The study was approved by the Research Ethics Committee of the Prefectural University of Hiroshima (letter no: 15MH070) and was conducted in accordance with the amended Declaration of Helsinki.

### Features

**Figure 7:**
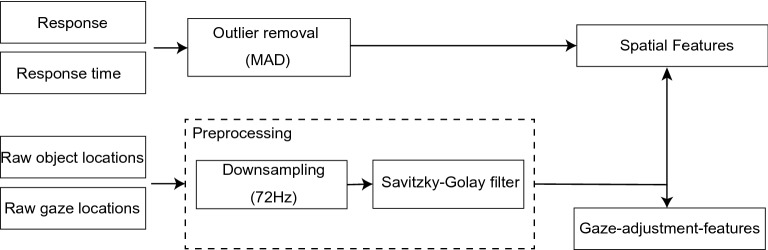
Features extraction pipeline used in this study. The inputs consists of gaze and object locations, response, and response time.

Figure 7 shows the pipeline for extracting spatial and gaze-adjustment features from response and gaze data. Before extracting the features, preprocessing was performed to eliminate noise and redundant data.

The responses whose RT was less than a threshold were considered as outliers; 6.6% of typical and 7.3% of ASD data were removed. The threshold was the RT’s median absolute deviation^30^ (104.75 ms) multiplied by a constant scale factor of the normal distribution (1.4826): 1.4826 $\times$ 104.75 = 155.30 ms. The data were down-sampled from 144 to 72 Hz to remove redundancy; a Savitzky-Golay filter^31^ ($n = 5$ and $poly = 2$) was used to perform smoothing to prevent artifacts during numerical differentiation.

#### Spatial features

The spatial features comprised 24 attributes (Table 6) that can be grouped into three categories: game performance, absolute gaze position, and gaze-to-object movement.

**Table 6:** The list of spatial features extracted from response and gaze behavior.

#	Detail
Go-positive	The percentage of Go response
Go-negative	The percentage of Go-negative response
NoGo-positive	The percentage of NoGo response
NoGo-negative	The percentage of NoGo-negative response
RT	The average of a subject's response time
RT-var	The standard deviation of a subject's response time
Trajectory-area	The gaze trajectory area
Velocity-avg	The average velocity of a subject's gaze
Velocity-var	The standard deviation of the velocity of a subject's gaze
Acceleration-avg	The average acceleration of a subject's gaze
Acceleration-var	The standard deviation of the velocity of a subject's gaze along the y-axis
Fixation-avg	The average of a subject's fixation time
Fixation-var	The standard deviation of a subject's fixation time
Distance-avg	The average of gaze distance
Distance-var	The standard deviation of gaze distance
Angle-avg	The average of gaze angle
Angle-var	The standard deviation of gaze angle
Distance-sen	Sample entropy of a subject's gaze distance
Angle-sen	Sample entropy of a subject's gaze angle
Velocity-sen	Sample entropy of gaze velocity
Spatial-en	The entropy of a subject's gaze distribution
Gaze-obj-en	The entropy of the difference between a subject's gaze and stimulus position
Gaze-obj-sen	Sample entropy of the Euclidean distance between a subject's gaze and stimulus position
Gaze-obj-spe	Spectral entropy of the difference between a subject's gaze and stimulus position

Average was computed with arithmetic mean and variance with standard deviation.

Game performance features consisted of subjects’ average response time (R_t_) and variance (R_t_-var) and the percentages of their positive and negative responses towards the Go and NoGo stimuli. R_t_ (Eq. 1) measured the time difference between when the character appeared ($t_{a}$) and when the subject reacted to it ($t_{r}$); while RT-var was the standard deviation of R_t_. The RT of the Go and NoGo stimuli were not separated.

1 $$\begin{aligned} R_t = t_{a} - t_{r} \end{aligned}$$

Gaze position features measured the absolute position of subjects’ gaze during the experiment. The gaze trajectory area was calculated using the Convex Hull algorithm^32^, and its value was normalized into 0 to 1. Gaze velocity and acceleration along the *x* and *y* axes were calculated respectively as the Savitzky-Golay smoothed first and second time-derivatives of the corresponding coordinate locations. Gaze distance (Eq. 4) and angles (Eq. 5) were the Euclidean distance and angle between gaze positions (*g*(*t*) and $g(t+1)$). The gaze spatial distribution was estimated using a 2D histogram algorithm, using 100 cells along each of the *x* and *y* axes.

2 $$v = \sqrt {\partial _{t} x^{2} + \partial _{t} y^{2} }$$

3 $$\partial v = \sqrt{{\partial _t ^ 2 x}^2 + {\partial _t ^ 2 y}^2}$$

4 $$d(t) = |g(t+1) - g(t)|$$

5 $$a(t) = \arccos \left( \frac{g(t+1) \cdot g(t)}{|g(t+1)|\cdot |g(t)|}\right)$$

Gaze-to-object movement comprised the features that computed the relative position between subjects’ gaze position and the stimulus position. Fixation time was measured as the time difference between when the subject’s gaze entered and when it left the stimulus area; the area was a circle of radius 0.25 (measured by Euclidean distance) from the center of the stimulus. Gaze-to-object difference was subjects’ gaze positions minus object positions when the latter appeared on the screen. Kernel density estimation and Welch’s method^33^ ($nperseg = 32$) were employed to estimate the probability and spectral densities of the difference, respectively. The probability distribution of the gaze-to-object difference was computed using 50 cells along each of the *x* and *y* axes.

Regularity of gaze distribution was computed using Shannon entropy^34^ and expressed as:

6 $$\begin{aligned} H_s = - \int p(x, y) \log _2(p(x, y)) dx dy \end{aligned}$$

where *p*(*x*, *y*) was the probability density of a state in a two-coordinate plane; while for power spectral density, *p*(*x*, *y*) was the sum of squared magnitudes of the Fourier transforms of the respective x and y components. Greater entropy indicates less intentional viewing behavior^28^ and suggests greater gaze dispersion. The final value of gaze entropy was normalized by diving by the maximum possible entropy $\log _2(N)$, in which *N* was the total number of states; in this study, N equaled to the total number of cells along the *x* and *y* axes.

Temporal randomness of gaze movement was measured by sample entropy (sen). Sen is equal to the negative natural logarithm of the probability that two subsequences of equal length *m* that are similar will remain similar at the next time step^35^; higher sample entropy means lower predictability within the original sequence. This study set *m* to two and estimated the distance between two template vectors with Chebyshev distance.

#### Gaze-adjustment features

Gaze-adjustment measured the distance between participants’ gaze and stimulus positions during times when the stimulus was presented onscreen. Euclidean distance was used as the distance metric, with values ranging from 0 to $\sqrt{2}$. Since the appearance time of each stimulus depended on the subjects’ RT, which varied, each gaze-adjustment was represented by auto-regressive parameters (Eq. 7). The model’s lag *L* was set to two (average of AIC: $-9.129$); hence, each gaze-adjustment was represented by three variables: $\alpha$, $\theta _2$, and $\theta _1$.

7 $$\begin{aligned} y_t = \alpha + \sum _{i=1}^{L} \theta _i y_{t-i} \end{aligned}$$

This study computed the average value of each coefficient for typical and ASD groups using the arithmetic means over the respective groups. Also, the average coefficients were calculated for each response type to investigate the difference between ASD and typical children’s gaze adjustment characteristics in more detail.

### Data analyses

#### Statistical analysis

The comparison between groups was computed using unpaired Student *t* and Mann-Whitney *U*^21^ tests. While multiple comparison was performed with ANOVA test. The effect size was calculated with Cohen’s *d*  ^36^; typical and ASD subjects were treated as the first and the second groups, respectively. The Benjamini-Hochberg procedure^37^ was utilized to control the false discovery rate associated with multiple comparisons, at the level 0.05.

#### Classification

Classification was performed to verify how informative the spatial and gaze-adjustment features are for differentiating ASD children from the typical ones. Instead of using raw gaze-adjustment features, this study computed the gaze-adjustment features’ skewness, kurtosis, average, and standard deviation. Then, the features’ dimension was reduced with Neighborhood Component Analysis^38^ and five components were retained; Principal Component Analysis was employed to initialize the transformation.

Spatial and gaze-adjustment features were normalized using *z*-normalization. The AdaBoost^22^ with decision tree base estimators was employed to distinguish the features of ASD children from those of typical children. The model’s hyper-parameter values were optimized using a grid search algorithm. In particular, this study validated the max-depth of the estimator in the set {1, 3, 5, 7}, the max-leaf of the estimator in {3, 5, 7}, the number of estimators in {15, 25, 50, 75}, and the AdaBoost’s learning-rate in {0.5, 0.75}. The max-feature-proportion of the decision tree was set to 0.5 for higher and lower proportion decreased the overall accuracy performance.

Accuracy, the Matthews Correlation Coefficient (MCC)^23^, and Area Under the Curve (AUC)^24^ were used as classification performance metrics. The decision threshold for classification was set to 0.5, thereby predicted probability greater or equal to 0.5 was converted to ASD class and typical class, otherwise. Three-fold cross-validation was used to validate the model.

Furthermore, this study examined the use of those features to identify ADHD symptoms in ASD children. The experiment utilized the same protocol as the classification of typical and ASD children.

## Supplementary Information

Supplementary Video 1.

Supplementary Video 2.

Supplementary Video 3.

Supplementary Information.
