# Publisher Correction: Identifying autism spectrum disorder symptoms using response and gaze behavior during the Go/NoGo game CatChicken

**DOI:** 10.1038/s41598-021-03444-z

**Published:** 2021-12-08

**Authors:** Prasetia Utama Putra, Keisuke Shima, Sergio A. Alvarez, Koji Shimatani

**Affiliations:** 1 Graduate School of Engineering, Yokohama National University, Yokohama, Japan; 2 Faculty of Engineering, Yokohama National University, Yokohama, Japan; 3 Department of Computer Science, Boston College, Chestnut Hill, USA; 4 Faculty of Health and Welfare, Prefectural University of Hiroshima, Hiroshima, Japan

Correction to: *Scientific Reports* 10.1038/s41598-021-01050-7, published online 10 November 2021

In the original version of this Article Keisuke Shima was incorrectly affiliated with ‘Graduate School of Engineering, Yokohama National University, Yokohama, Japan’. The correct affiliation is listed below.

Faculty of Engineering, Yokohama National University, Yokohama, Japan.

The original Article has been corrected.

