# Supplementary material for: Identifying autism spectrum disorder symptoms using response and gaze behavior during the Go/NoGo game CatChicken
Source: Sci Rep. 2021 Nov 10;11:22012. doi: 10.1038/s41598-021-01050-7 (PMC8581032; doi:10.1038/s41598-021-01050-7)
Supplement: Supplementary file 4 — Supplementary Information. [file 41598_2021_1050_MOESM4_ESM.pdf]

Supplementary Materials

Identifying Autism Spectrum Disorder Symptoms Using Response and Gaze Behavior during the Go/NoGo Game CatChicken

Prasetia Utama Putra, Keisuke Shima, Sergio A. Alvarez, Koji Shimatani

1 CatChicken game architecture

CatChicken game is a serious game based on a Go/NoGo task that can be used to measure children's response and gaze behavior during the task. The system comprises game and results interfaces (Fig. S1). The game interface was developed on the Unity game platform and uses characters developed by VoxelGuy that can be accessed from the Unity store (5 animated Voxel animals: `https://assetstore.unity.com/packages/3d/characters/animals/5-animated-voxel-animals-145754`).

The game represents Go and NoGo stimuli as "cat" and "chicken" characters, respectively. The game requires the subject to respond to a "chicken" character by pressing a spacebar and inhibit their action towards a "cat" character. A character can appear in one of nine locations represented by red flowers.

The CatChicken system comprises two main functions: training and evaluation. Using the training function, an instructor can modify the game's parameters that include the task duration, the appearance time of a stimulus, the proposition of stimuli, the interval between two consecutive stimuli, and the locations at which a stimulus appears. While using the evaluation function, those parameters can be fixed to create a standardized task for all subjects.

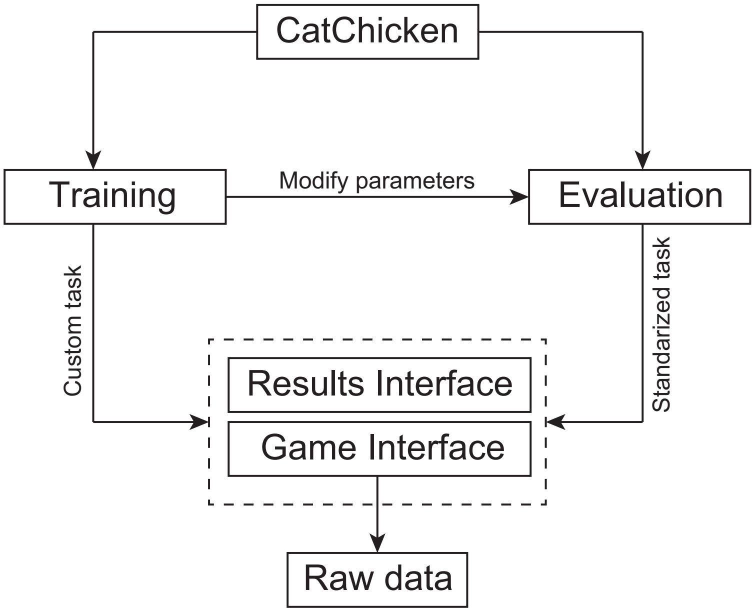

Figure S1: Architecture of the CatChicken system. The game produces raw data that consist of the subject's response types and times, and locations of stimulus and gaze over time.

After a subject finishes playing the game, their results are shown in the results interface. The results interface shows a subject's game performance in the form of response time, its variance, and the percentages of correct, incorrect, and miss responses; Go-positive and NoGo-positive are correct responses; NoGo-negative is an incorrect response, and Go-negative is a miss response.

The CatChicken game was developed by utilizing the following assets from Unity Store:

1. Bolt Kit: First Person
`https://assetstore.unity.com/packages/templates/tutorials/bolt-kit-first-person-149467`

2. 5 animated Voxel animals by VoxelGuy
`https://assetstore.unity.com/packages/3d/characters/animals/5-animated-voxel-animals-145754`

3. Effect textures and prefabs
`https://assetstore.unity.com/packages/vfx/particles/effect-textures-and-prefabs-109031`

CatChicken is available at `https://sourceforge.net/projects/catchicken/`

2 Subject information

No	Code	Sex	Label	Note
1	AT_000013	F	Typical	Excluded
2	AT_000014	F	Typical	
3	AT_000015	F	Typical	
4	AT_000016	F	Typical	Excluded
5	AT_000018	M	Typical	
6	AT_000019	M	Typical	
7	AT_000020	M	Typical	
8	AT_000021	M	Typical	Excluded
9	AT_000022	M	Typical	
10	AT_000024	F	Typical	
11	AT_000025	F	Typical	
12	AT_000026	M	Typical	
13	AT_000028	M	Typical	
14	AT_000029	M	Typical	
15	AT_000030	M	Typical	
16	AT_000031	M	Typical	
17	AT_000033	M	Typical	
18	AT_000034	M	Typical	
19	AT_000035	M	Typical	
20	AT_000036	M	Typical	
21	AT_000037	M	Typical	
22	AT_000038	M	Typical	
23	AT_000040	M	Typical	
24	AT_000041	M	Typical	
25	AT_000042	F	Typical	
26	AT_000043	F	Typical	
27	AT_000044	F	Typical	
28	AT_000046	F	Typical	
29	AT_000047	F	Typical	
30	AT_000048	M	Typical	
31	AT_000049	M	Typical	
32	AT_000050	M	Typical	
33	AT_000051	F	Typical	Excluded
34	AT_000052	M	Typical	
35	AT_000053	F	Typical	

Table S1: List of typical subjects.

No	Code	Sex	Label	Note
1	HT_000005	M	ASD	ADHD
2	HT_000006	M	ASD	
3	HT_000007	M	ASD	
4	HT_000008	F	ASD	ADHD
5	HT_000009	F	ASD	ADHD
6	HT_000010	M	ASD	
7	HT_000011	M	ASD	
8	HT_000012	M	ASD	ADHD
9	HT_000013	M	ASD	
10	HT_000014	M	ASD	ADHD
11	HT_000015	F	ASD	
12	HT_000016	F	ASD	
13	HT_000017	M	ASD	ADHD
14	HT_000018	M	ASD	Excluded
15	HT_000019	M	ASD	ADHD
16	HT_000020	F	ASD	ADHD
17	HT_000021	M	ASD	
18	HT_000022	F	ASD	
19	HT_000023	M	ASD	
20	HT_000024	M	ASD	ADHD suspected (we ignored the ADHD label and assumed this subject as ASD only)
21	HT_000026	M	ASD	ADHD
22	HT_000027	M	ASD	ADHD

Table S2: List of subjects with Autism Spectrum Disorder (ASD). ADHD stand for Attention Deficit Hyperactive Disorder comorbidities.

3 Statistical analysis of spatial features

	Typical (Mean ± STD)	ASD (Mean ± STD)	ASD without ADHD (Mean ± STD)	ASD with ADHD (Mean ± STD)
Go-positive	30.8 ± 9.9%	24.2 ± 12.9%	20.8 ± 10.5%	27.9 ± 14.8%
Go-negative	19.0 ± 10.0%	25.7 ± 12.9%	29.3 ± 10.7%	21.7 ± 14.4%
NoGo-positive	46.2 ± 2.7%	45.9 ± 3.4%	45.7 ± 3.0%	46.1 ± 4.0%
NoGo-negative	4.0 ± 2.8%	4.2 ± 3.0%	4.2 ± 2.7%	4.2 ± 3.5%
RT	545 ± 34.3	539 ± 48.7	529 ± 56.2	551 ± 38.6
RT-var	117 ± 36.9	136 ± 35.7	146 ± 31.7	126 ± 38.3
Trajectory-area	0.521 ± 0.103	0.515 ± 0.134	0.501 ± 0.109	0.530 ± 0.161
Velocity-avg	0.0088 ± 0.0010	0.0098 ± 0.0024	0.0093 ± 0.0011	0.0103 ± 0.0034
Velocity-var	0.0176 ± 0.0024	0.0198 ± 0.0051	0.0186 ± 0.0025	0.0212 ± 0.0069
Acceleration-avg	0.0035 ± 0.0006	0.0044 ± 0.0016	0.0040 ± 0.0007	0.0047 ± 0.0022
Acceleration-var	0.0076 ± 0.0015	0.0092 ± 0.0034	0.0085 ± 0.0017	0.0101 ± 0.0046
Fixation-avg	405 ± 20.6	399 ± 28.9	409 ± 12.6	389 ± 38.0
Fixation-var	174 ± 8.2	183 ± 8.3	182 ± 8.9	183 ± 8.1
Distance-avg	0.0160 ± 0.0015	0.0171 ± 0.0035	0.0164 ± 0.0016	0.0178 ± 0.0049
Distance-var	0.0293 ± 0.0033	0.0316 ± 0.0063	0.0301 ± 0.0031	0.0333 ± 0.0084
Angle-avg	0.0130 ± 0.0013	0.0136 ± 0.0026	0.0131 ± 0.0011	0.0141 ± 0.0036
Angle-var	0.0285 ± 0.0039	0.0304 ± 0.0073	0.0285 ± 0.0036	0.0323 ± 0.0098
Distance-sen	0.147 ± 0.031	0.202 ± 0.076	0.207 ± 0.083	0.196 ± 0.072
Angle-sen	0.124 ± 0.026	0.169 ± 0.063	0.173 ± 0.063	0.165 ± 0.065
Velocity-sen	0.137 ± 0.034	0.200 ± 0.077	0.206 ± 0.088	0.193 ± 0.069
Spatial-en	0.894 ± 0.011	0.894 ± 0.020	0.895 ± 0.016	0.893 ± 0.024
Gaze-obj-en	0.484 ± 0.013	0.497 ± 0.017	0.494 ± 0.015	0.499 ± 0.020
Gaze-obj-sen	0.130 ± 0.018	0.153 ± 0.031	0.150 ± 0.027	0.158 ± 0.036
Gaze-obj-spe	0.354 ± 0.008	0.361 ± 0.006	0.360 ± 0.005	0.361 ± 0.008

Table S3: The average and standard deviation (STD) values of each spatial features for typical, ASD, ASD without ADHD, and ASD with ADHD groups. Percentage scale is used to express Go positive and negative, and NoGo positive and negative. While, RT, RT-var, fixation-avg, and fixation-var are expressed in millisecond.

	TYP vs ASD (d)	TYP vs ASD without ADHD (d)	TYP vs ASD with ADHD (d)	ASD without ADHD vs ASD with ADHD (d)
Go-positive	0.591	0.998	0.258	-0.559
Go-negative	-0.598	-1.014	-0.247	0.600
NoGo-positive	0.105	0.193	0.024	-0.134
NoGo-negative	-0.073	-0.081	-0.066	0.009
RT	0.151	0.402	-0.146	-0.443
RT-var	-0.518	-0.800	-0.221	0.581
Trajectory-area	0.056	0.196	-0.075	-0.216
Velocity-avg	-0.531	-0.431	-0.791	-0.417
Velocity-var	-0.589	-0.404	-0.902	-0.506
Acceleration-avg	-0.763	-0.783	-1.042	-0.457
Acceleration-var	-0.668	-0.556	-0.964	-0.478
Fixation-avg	0.229	-0.213	0.626	0.726
Fixation-var	-0.997	-0.883	-1.119	-0.208
Distance-avg	-0.451	-0.302	-0.699	-0.401
Distance-var	-0.482	-0.237	-0.789	-0.506
Angle-avg	-0.301	-0.094	-0.522	-0.372
Angle-var	-0.335	-0.010	-0.662	-0.525
Distance-sen	-1.026	-1.218	-1.129	0.133
Angle-sen	-1.020	-1.280	-1.054	0.136
Velocity-sen	-1.133	-1.307	-1.267	0.160
Spatial-en	-0.002	-0.069	0.059	0.087
Gaze-obj-en	-0.864	-0.787	-1.035	-0.265
Gaze-obj-sen	-0.973	-0.953	-1.184	-0.255
Gaze-obj-spe	-0.880	-0.833	-0.875	-0.128

Table S4: d denotes Cohen's effect size measure of spatial features between groups. TYP vs ASD means that typical (TYP) and ASD population were treated as the first and second group, respectively.

	TYP vs ASD		TYP vs ASD without ADHD		TYP vs ASD with ADHD		ASD without ADHD vs ASD with ADHD		ANOVA (p)
	Student (p)	Whitney (p)	Student (p)	Whitney (p)	Student (p)	Whitney (p)	Student (p)	Whitney (p)	
Go-positive	0.042	0.040	***0.007**	***0.005**	0.483	0.458	0.216	0.085	0.099
Go-negative	0.039	0.033	***0.006**	***0.005**	0.501	0.392	0.186	0.074	0.096
NoGo-positive	0.712	0.404	0.585	0.313	0.948	0.458	0.763	0.275	0.647
NoGo-negative	0.797	0.411	0.818	0.376	0.856	0.494	0.984	0.376	0.596
RT	0.595	0.334	0.259	0.284	0.690	0.470	0.323	0.349	0.857
RT-var	0.073	0.032	0.028	***0.012**	0.547	0.277	0.200	0.123	0.197
Trajectory-area	0.845	0.321	0.580	0.344	0.837	0.375	0.626	0.349	0.124
Velocity-avg	0.066	0.102	0.227	0.139	0.036	0.185	0.352	0.486	***0.006**
Velocity-var	0.042	0.070	0.257	0.120	0.018	0.127	0.261	0.458	***0.007**
Acceleration-avg	***0.009**	***0.002**	0.031	***0.008**	***0.007**	0.020	0.309	0.486	***0.001**
Acceleration-var	0.022	0.035	0.121	0.058	***0.012**	0.104	0.288	0.486	***0.003**
Fixation-avg	0.422	0.404	0.547	0.195	0.093	0.093	0.113	0.096	0.254
Fixation-var	***0.001**	***0.001**	***0.016**	***0.010**	***0.004**	***0.002**	0.639	0.275	***0.004**
Distance-avg	0.117	0.234	0.395	0.237	0.062	0.341	0.370	0.458	***0.013**
Distance-var	0.095	0.136	0.503	0.246	0.036	0.147	0.261	0.349	***0.025**
Angle-avg	0.292	0.500	0.791	0.454	0.159	0.446	0.405	0.458	0.034
Angle-var	0.241	0.341	0.978	0.500	0.076	0.247	0.244	0.299	0.062
Distance-sen	***0.001**	***0.001**	***0.001**	***0.006**	***0.004**	***0.011**	0.765	0.403	***0.001**
Angle-sen	***0.001**	***0.001**	***0.001**	***0.004**	***0.006**	0.019	0.760	0.275	***0.001**
Velocity-sen	***0.000**	***0.000**	***0.001**	***0.004**	***0.001**	***0.002**	0.718	0.430	***0.000**
Spatial-en	0.993	0.195	0.845	0.115	0.873	0.458	0.845	0.230	0.223
Gaze-obj-en	***0.004**	***0.004**	0.030	0.031	***0.007**	***0.011**	0.552	0.349	***0.006**
Gaze-obj-sen	***0.001**	***0.001**	***0.010**	***0.016**	***0.002**	***0.002**	0.566	0.458	***0.001**
Gaze-obj-spe	***0.003**	***0.002**	0.023	***0.010**	0.021	0.025	0.772	0.376	***0.013**

Table S5: Statistical significance between-group (p) differences of individual spatial features, as measured by Student t, Mann-Whitney U, and ANOVA tests.TYP vs ASD means that typical (TYP) and ASD population were treated as the first and second group, respectively. * indicates significant p-value after controlling false discovery rate at level 0.05.

4 Statistical analysis of gaze adjustment features

	Typical (Mean ± STD)	ASD (Mean ± STD)	ASD without ADHD (Mean ± STD)	ASD with ADHD (Mean ± STD)
Average				
C1	0.004 ± 0.012	0.005 ± 0.013	0.005 ± 0.015	0.005 ± 0.010
C2	1.488 ± 0.221	1.456 ± 0.189	1.446 ± 0.179	1.467 ± 0.199
C3	-0.520 ± 0.206	-0.489 ± 0.191	-0.475 ± 0.176	-0.503 ± 0.206
Go-positive				
C1	0.002 ± 0.009	0.005 ± 0.007	0.004 ± 0.006	0.006 ± 0.008
C2	1.533 ± 0.060	1.488 ± 0.167	1.455 ± 0.098	1.525 ± 0.213
C3	-0.561 ± 0.054	-0.529 ± 0.193	-0.487 ± 0.095	-0.575 ± 0.254
Go-negative				
C1	0.003 ± 0.002	0.002 ± 0.005	0.001 ± 0.004	0.003 ± 0.005
C2	1.572 ± 0.059	1.521 ± 0.084	1.536 ± 0.063	1.505 ± 0.099
C3	-0.601 ± 0.054	-0.549 ± 0.077	-0.560 ± 0.058	-0.536 ± 0.092
NoGo-positive				
C1	0.003 ± 0.002	0.005 ± 0.008	0.003 ± 0.003	0.007 ± 0.011
C2	1.606 ± 0.031	1.540 ± 0.081	1.555 ± 0.059	1.523 ± 0.097
C3	-0.631 ± 0.028	-0.571 ± 0.066	-0.583 ± 0.050	-0.558 ± 0.077
NoGo-negative				
C1	0.010 ± 0.021	0.009 ± 0.023	0.011 ± 0.029	0.006 ± 0.012
C2	1.241 ± 0.321	1.274 ± 0.238	1.238 ± 0.218	1.314 ± 0.252
C3	-0.286 ± 0.295	-0.306 ± 0.230	-0.272 ± 0.219	-0.344 ± 0.236

Table S6: The average and standard deviation (STD) values of the gaze-adjustment features for typical, ASD, ASD without ADHD, and ASD with ADHD groups. α, θ_1, and θ_2 are the auto-regressive model's constant term, first, and second coefficients, respectively.

	TYP vs ASD (d)	TYP vs ASD without ADHD (d)	TYP vs ASD with ADHD (d)	ASD without ADHD vs ASD with ADHD (d)
Average				
C1	-0.065	-0.056	-0.077	-0.012
C2	0.153	0.198	0.097	-0.109
C3	-0.156	-0.223	-0.080	0.145
Go-positive				
C1	-0.385	-0.287	-0.462	-0.225
C2	0.379	1.061	0.064	-0.414
C3	-0.245	-1.081	0.103	0.449
Go-negative				
C1	0.362	0.659	0.164	-0.289
C2	0.703	0.581	0.909	0.350
C3	-0.807	-0.740	-0.974	-0.294
NoGo-positive				
C1	-0.429	-0.336	-0.698	-0.390
C2	1.155	1.246	1.493	0.394
C3	-1.259	-1.340	-1.589	-0.377
NoGo-negative				
C1	0.044	-0.072	0.199	0.241
C2	-0.114	0.007	-0.234	-0.306
C3	0.074	-0.049	0.202	0.302

Table S7: d denotes Cohen's effect size measure of gaze-adjustment features between groups. TYP vs ASD means that typical (TYP) and ASD population were treated as the first and second group, respectively.

	TYP vs ASD		TYP vs ASD without ADHD		TYP vs ASD with ADHD		ASD without ADHD vs ASD with ADHD		ANOVA (p)
	Student (p)	Whitney (p)	Student (p)	Whitney (p)	Student (p)	Whitney (p)	Student (p)	Whitney (p)	
Average									
C1	0.646	***0.000**	0.750	***0.000**	0.671	***0.000**	0.956	***0.000**	0.899
C2	0.282	***0.023**	0.262	***0.000**	0.594	***0.023**	0.620	***0.023**	0.506
C3	0.272	***0.000**	0.205	***0.000**	0.660	***0.000**	0.509	***0.000**	0.448
Go-positive									
C1	0.180	***0.000**	0.418	***0.000**	0.212	***0.000**	0.613	***0.000**	0.370
C2	0.185	***0.023**	***0.004**	***0.000**	0.862	***0.023**	0.356	0.023	0.164
C3	0.391	***0.000**	***0.004**	***0.000**	0.778	***0.000**	0.317	***0.000**	0.213
Go-negative									
C1	0.206	***0.000**	0.068	***0.000**	0.654	***0.000**	0.516	***0.000**	0.295
C2	0.016	***0.000**	0.106	***0.023**	***0.017**	***0.000**	0.433	***0.000**	0.036
C3	***0.006**	***0.000**	0.041	***0.000**	***0.011**	***0.000**	0.509	***0.000**	0.018
NoGo-positive									
C1	0.135	***0.000**	0.344	***0.000**	0.062	***0.000**	0.383	***0.000**	0.130
C2	***0.000**	***0.023**	***0.001**	***0.023**	***0.000**	***0.023**	0.378	***0.023**	***0.000**
C3	***0.000**	***0.000**	***0.000**	***0.000**	***0.000**	***0.000**	0.399	***0.000**	***0.000**
NoGo-negative									
C1	0.877	***0.000**	0.838	***0.000**	0.586	***0.000**	0.587	***0.000**	0.834
C2	0.688	***0.000**	0.984	***0.000**	0.524	***0.000**	0.493	***0.023**	0.781
C3	0.794	***0.000**	0.890	***0.000**	0.582	***0.000**	0.498	***0.000**	0.811

Table S8: Statistical significance between-group (p) differences of individual gaze-adjustment features, as measured by Student t, Mann-Whitney U, and ANOVA tests. TYP vs ASD means that typical (TYP) and ASD population were treated as the first and second group, respectively. * indicates significant p-value after controlling false discovery rate at level 0.05.

5 Cross-validation

This study validated the performance of the proposed model with three-fold cross-validation. In each cross-validation fold, 7 ASD and 10 to 11 typical children were used as test set and the rest as training set.

1st-Fold	2nd-Fold	3rd-Fold
AT_000014	AT_000018	AT_000015
AT_000019	AT_000025	AT_000020
AT_000029	AT_000026	AT_000022
AT_000031	AT_000030	AT_000024
AT_000036	AT_000033	AT_000028
AT_000038	AT_000035	AT_000034
AT_000040	AT_000041	AT_000037
AT_000044	AT_000043	AT_000042
AT_000046	AT_000052	AT_000047
AT_000048	AT_000053	AT_000050
AT_000049		

Table S9: List of typical children included in the test set in the first, second, and third folds.

1st-Fold	2nd-Fold	3rd-Fold
HT_000008	HT_000006	HT_000013
HT_000009	HT_000007	HT_000015
HT_000011	HT_000010	HT_000020
HT_000014	HT_000016	HT_000022
HT_000019	HT_000017	HT_000026
HT_000023	HT_000021	HT_000027
HT_000024	HT_000005	HT_000012

Table S10: List of children with ASD included in the test set in the first, second, and third folds.

6 Response time

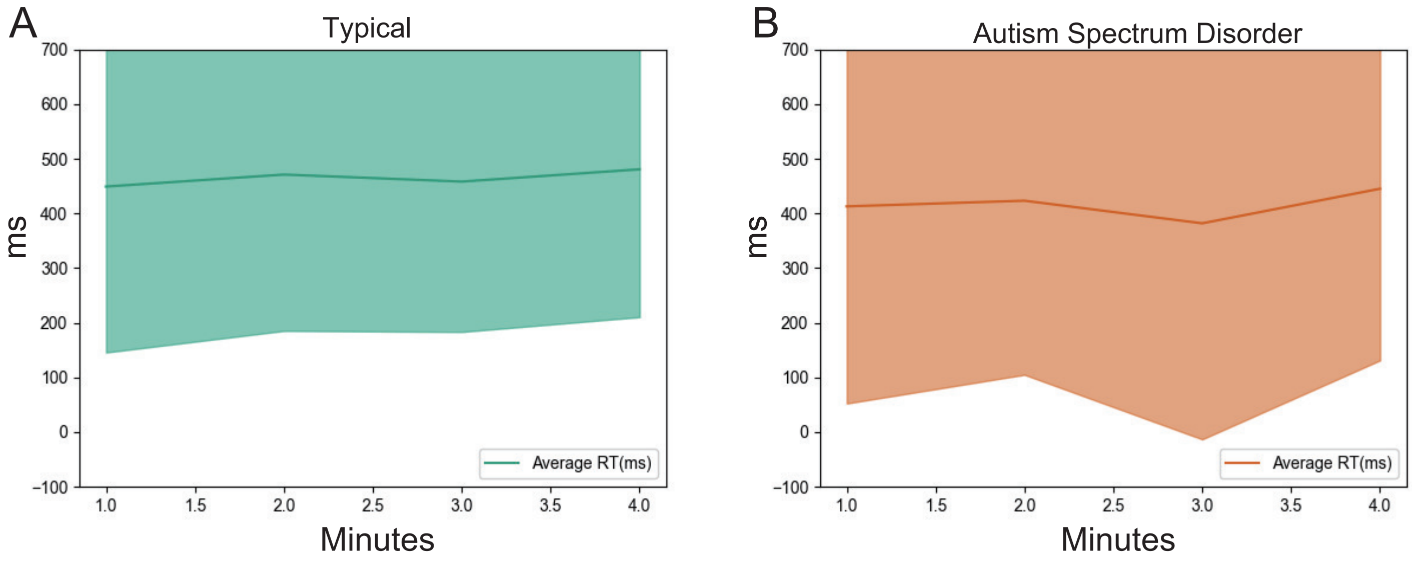

Figure S2: Average response time of typical (A) and ASD (B) children. Solid line indicates the average and shading represents the standard deviation

7 Gaze trajectory

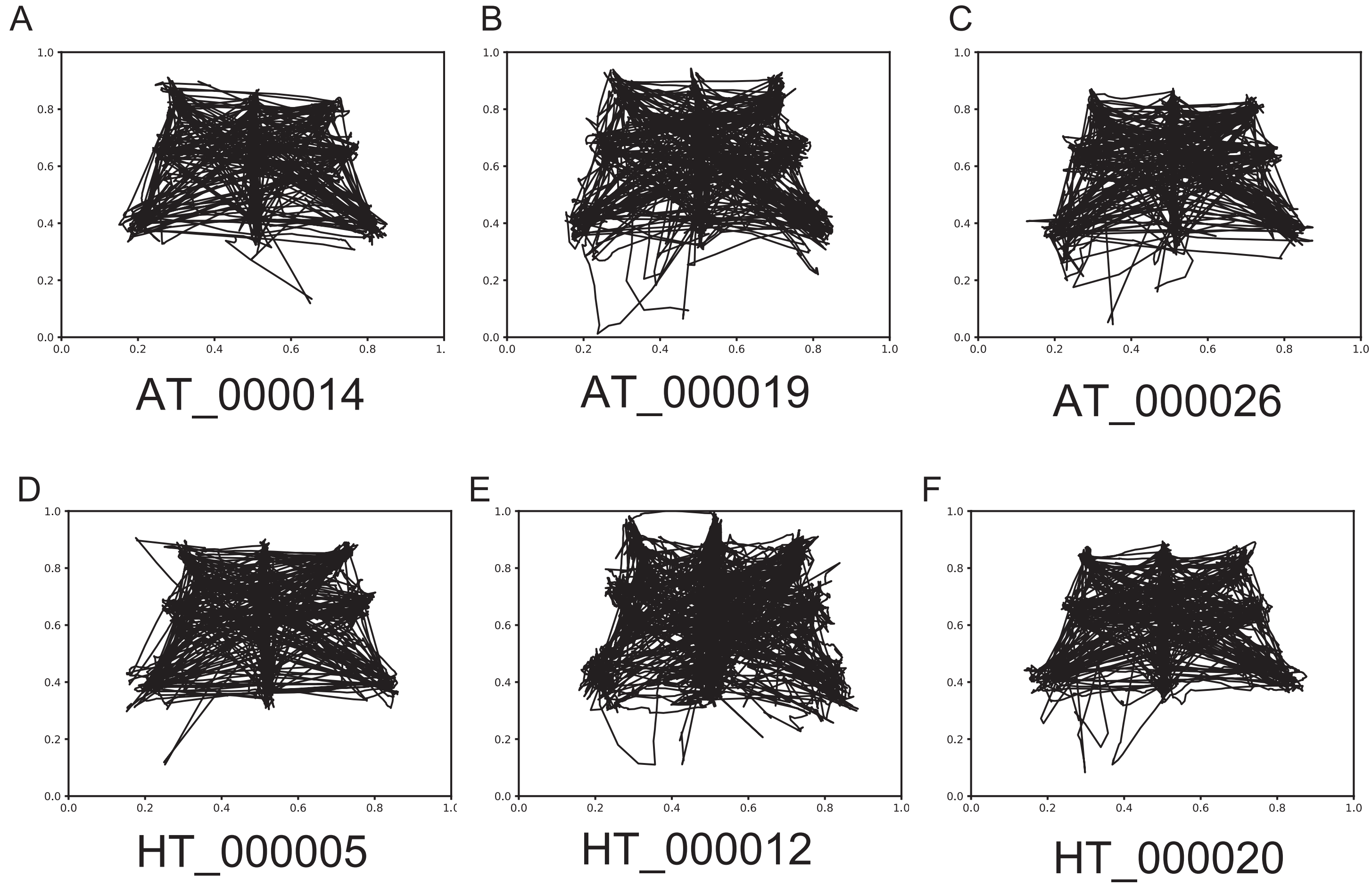

Figure S3: (**A - C**) Samples of typical subjects' gaze trajectories. (**D - F**) Samples of ASD subjects' gaze trajectories.

8 Gaze acceleration

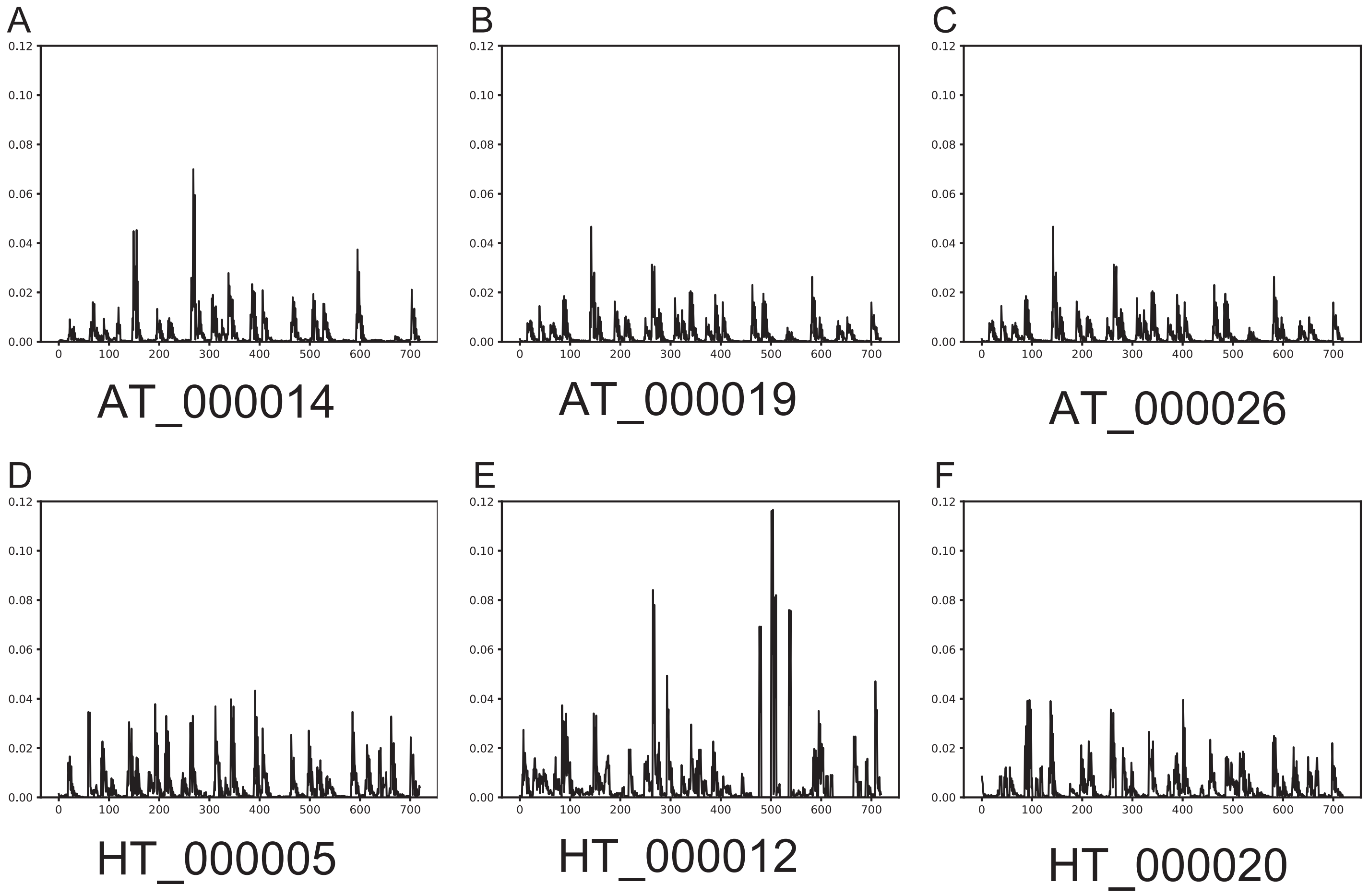

Figure S4: (**A - C**) Samples of typical subjects' gaze acceleration. (**D - F**) Samples of ASD subjects' gaze acceleration.

9 Gaze-to-object-difference density

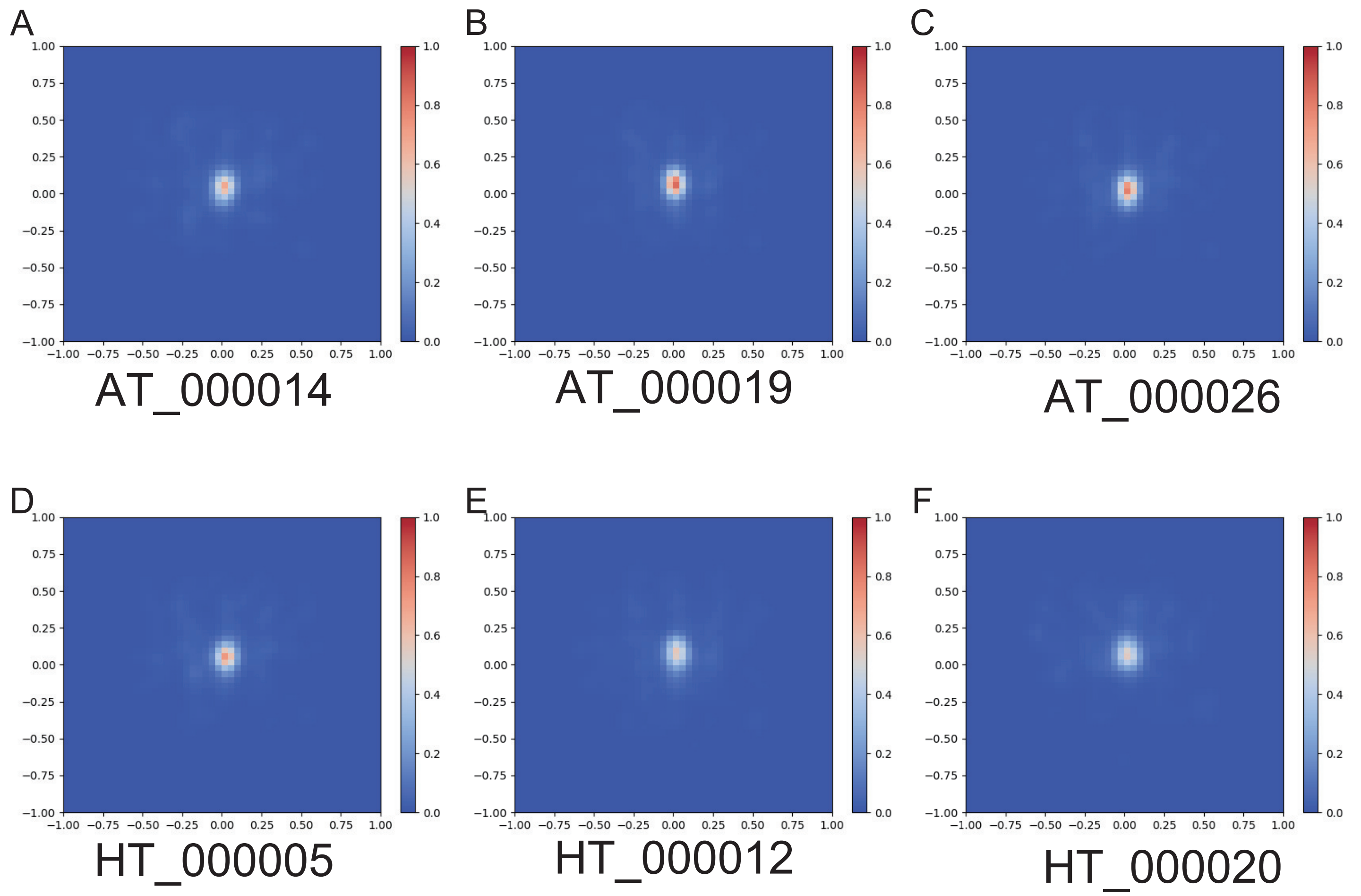

Figure S5: (**A - C**) Samples of typical subjects' gaze-to-object-difference density. (**D - F**) Samples of ASD subjects' gaze-to-object-difference density.

10 Data and source code

https://osf.io/4hrqx/

11 Videos

Video S1. A demonstration of CatChicken game with an adult as the subject. For better visualization the application was run on a desktop PC with a monitor (AttentionTest.mp4).
Video S2. User interface of CatChicken game. Audio feedback was given when the subject reacts correctly or incorrectly (AttentionTestFinal_nomark.mp4).
Video S3. Replay of subjects' gaze and stimulus position during the experiment. AT_000014 and HT_000012 are typical and ASD children, respectively (Gaze_1.mp4). Blue and red cross represent the subject's gaze and stimulus positions, respectively.
